# Individual Antipredator Responses Are Positively Correlated Across Cue Types in Free‐Living Black‐Capped Chickadees ( *Poecile atricapillus* )

**DOI:** 10.1002/ece3.72016

**Published:** 2025-08-17

**Authors:** Emma L. C. Reid, Megan LaRocque, Josue David Arteaga‐Torres, Kimberley J. Mathot

**Affiliations:** ^1^ Department of Biological Sciences University of Alberta Edmonton Alberta Canada; ^2^ Canada Research Chair in Integrative Ecology, Department of Biological Sciences University of Alberta Edmonton Alberta Canada

**Keywords:** antipredatory response, black‐capped chickadees, cue integration, foraging behavior, information theory

## Abstract

Prey animals must accurately assess predation risk within their environment. To gather information about this risk, prey animals may personally sample the environment (“personal information”) or observe the behavior of congeners (“social information”). Personal information is thought to be more accurate and reliable but may also require more time and energy to acquire. On the other hand, social information, such as alarm calls, tends to be less costly to obtain but may also be less reliable if congeners assess risk differently from one another, or if the information quickly becomes outdated. Theoretical models predict that individuals will differ in how they value personal versus social information. We used previously collected data from a marked population of black‐capped chickadees ( *Poecile atricapillus* ) to test this prediction. Chickadees were exposed to three different predator cue types: a predator mount (personal information), conspecific mobbing calls (social information), and a combination of both (personal + social information) near feeders established on their territory. We recorded the time it took a chickadee to visit a feeder following cue exposure (i.e., latency to resume feeding) to evaluate individual differences in response to predator cues. Contrary to our prediction, we found no evidence that individuals differed in how they valued personal versus social information about predation risk. Instead, our results suggest that responses to predator cues are state‐dependent, with some individuals consistently responding more strongly than others, regardless of cue type. We also found that when chickadees were exposed to a combination of social and personal predator cues, they exhibited higher among‐individual variation in latency to resume feeding than when they were exposed to social or personal cues alone. We discuss how individual differences in cue integration (i.e., cue redundancy/equivalence, enhancement, and antagonism) may account for this finding.

## Graphical abstract

Chickadees can use different sources of information (e.g., personal vs. social) to evaluate predation risk. Individual responses to different sources of information were positively correlated; birds that responded strongly to personal information also responded strongly to social. Combined cues led to higher variation among individuals, suggesting that individuals differ in how they integrate social versus personal information cues.

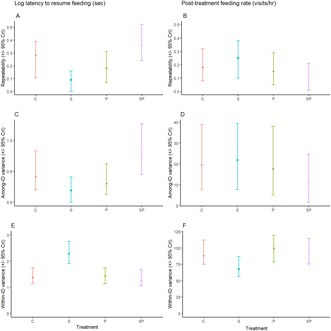

## 1 Introduction

The ability to accurately assess and appropriately respond to the threat of predation is essential, and there is a large body of empirical work investigating how prey animals respond to variation in predation risk (reviewed in Crane et al. 2024; Griffin 2004; Jones et al. 2024; Lima and Dill 1990; Mathot et al. 2024). Studies have used a variety of cue types to experimentally manipulate perceived predation risk, and these cues often provide different types of information to organisms. For example, presenting a model predator (Webster and Laland 2008) or playbacks of predator calls (Carlson et al. 2017) provides individuals with direct information about predator presence, forming “personal information” (Danchin et al. 2004). On the other hand, conspecific behavior (Wilson et al. 2021), distress chemicals (Brown et al. 2006), and alarm calls (Templeton et al. 2005) inform an individual that congeners perceive risk within the environment. These conspecific cues indirectly inform an individual of predation risk and are forms of “social information” (Danchin et al. 2004). Furthermore, some studies manipulate personal and social cues of predation risk separately and simultaneously and then assess population‐level responses (e.g., Arteaga‐Torres et al. 2020; Conover and Perito 1981; Desrochers et al. 2002). Together, these studies provide an interesting paradigm to test how individuals within the population might differ in how they value personal versus social information about predation risk.

The extent to which individuals invest in and respond to personal versus social information is expected to be context‐dependent (Danchin et al. 2004; Jones et al. 2024; Kendal et al. 2005; Laland 2004; Rieucau and Giraldeau 2011). Personal information tends to be more reliable but is costly to acquire, whereas social information is relatively inexpensive but may be less accurate (Kendal et al. 2005; Rauber and Manser 2018; Rieucau and Giraldeau 2011). Various strategies have been proposed for how individuals might balance these costs and benefits. For example, under a “copy‐when‐asocial‐learning‐is‐costly” strategy (reviewed in Laland 2004), individuals will generally shift toward favoring social information as the cost of acquiring personal information increases (reviewed in Kendal et al. 2005). For example, minnows ( *Phoxinus phoxinus* ) were more likely to ignore their personal experience and copy demonstrators when perceived predation risk was higher, making the acquisition of personal information relatively more costly (Webster and Laland 2008). However, individuals may differ in the way that they respond to social versus personal information within a given context. Repeatable among‐individual differences in relative preference for social versus personal information have been documented in foraging (reviewed in Rieucau and Giraldeau 2011) and nest‐building (Whittaker et al. 2023) contexts, and some studies have documented sex‐related differences in the relative collection of personal versus social information (e.g., Schuett and Dall 2009). To date, investigation of repeatable among‐individual variation in response to social versus personal cues of predation risk is lacking. However, given that among‐individual differences in vulnerability to predation are well documented (reviewed in Mesa et al. 1994; Pettorelli et al. 2011), it seems likely that individuals should also differ in the costs and benefits of acquiring and responding to personal versus social information about that risk.

Here, we re‐analyse data from a previously published study that manipulated both personal and social cues of predation risk in a wild population of black‐capped chickadees ( *Poecile atricapillus* ) (Arteaga‐Torres et al. 2020). Chickadees form stable winter flocks characterized by social foraging and shared vigilance (Smith 1991), making them an excellent study organism for investigating individual differences in response to personal versus social information about predation risk. Subjects were presented with (i) audio playbacks of chickadee mobbing calls (social information), (ii) stuffed merlin mounts ( *Falco columbarius* ; personal information), and (iii) combined treatments including both mobbing calls and a merlin mount (social + personal information). Response to perceived predation was measured as the time it took a chickadee to revisit a feeder after exposure to a predator cue (i.e., latency to resume feeding), and the number of visits they made to the feeder in the hour following their return (i.e., post‐treatment feeding rate). At the population level, latency to resume feeding was longer in treatments involving the stuffed merlin (Arteaga‐Torres et al. 2020), suggesting that chickadees respond more strongly to personal information about predation risk. However, repeatable among‐individual differences in response across all treatments were also observed (Arteaga‐Torres et al. 2020). Here, we evaluated whether chickadees show among‐individual differences in their prioritization of personal versus social information. If they do, we predicted that individuals who have a stronger response to the social treatment would have a weaker response to the personal treatment (and vice versa), and that the correlation of individual responses between treatments that share information types (personal and social + personal, as well as social and social + personal), would be stronger compared to the correlation between treatments that do not share information types (personal vs. social). We additionally evaluate treatment‐related differences in within‐ and among‐individual variance in response to different cue types based on a recent meta‐analysis suggesting that higher cue uncertainty may be associated with higher within‐individual variance in response (Mathot et al. 2024). Our results contribute to a growing body of work that aims to shed light on how cues are integrated in risk assessment.

## 2 Methods

### 2.1 Study Site and Study Population

This study was conducted on a marked population of black‐capped chickadees (henceforth “chickadees”) at the University of Alberta Botanic Garden (UABG; 53°24′27″ N, 113°45′41″ W) from late November 2018 to early March 2019. The UABG is a 0.97 km^2^ property with 0.32 km^2^ of display gardens and 0.65 km^2^ of mixed wood forest. Between October and November of 2017 and 2018, chickadees were caught with mist nets set up near eight feeders filled with black oil sunflower seeds. Any birds not previously caught were fitted with Canadian Wildlife Service aluminium leg bands and colored leg bands for personal recognition. Immediately after banding, birds underwent two short standardized behavioral assays as part of standard data collection in this population. Chickadees were then measured for standard morphometrics (e.g., tarsus length, bill length, and bill depth) and body mass, and had a small blood sample collected for molecular sexing (Griffiths et al. 1998).

### 2.2 Experimental Setup and Data Collection

Data collected were originally used to evaluate population‐level responses to different cues of predation risk (Arteaga‐Torres et al. 2020). Here, we present a re‐analysis of those data focused on individual‐level responses. Detailed descriptions of the experimental setup and data collection are presented in Arteaga‐Torres et al. (2020), but we provide a brief overview here. Eight feeders, spaced at least 270 m apart, were placed throughout the UABG. Feeders were baited with black oil sunflower seeds and equipped with a radio frequency identification antenna, positioned at the feeder opening. Daily mean temperature was taken from the Edmonton International Airport (YEG) weather station, 10 km SE of the study site (data acquired from Alberta Agriculture and Forestry, ACIS: https://agriculture.alberta.ca/acis).

We used a 2 × 2 factorial design to combine our stimuli into four treatments: (i) audio playback alone (social information), (ii) a merlin mount alone (personal information), (iii) audio playback and a merlin mount (social + personal information), and (iv) control. The social treatment consisted of a 1 h audio file made up of alternating mobbing calls (1–4 birds calling for 5–20 s, repeated to span a 1 min duration) and bouts of silence (60–180 s duration), which we played over speakers (Shockwave, Foxpro, Lewistown, PA) placed on a pole 3 m from the focal feeder. A total of 8 unique 1‐h sound files were used for the experiments. The personal treatment consisted of a merlin mount which we placed on a pole 3 m from the focal feeder. A total of 6 unique merlin mounts were used in the experiments. The control treatment provided a control for the non‐biological components of the experimental design in two ways. First, we kept speakers on the poles for the duration of all experimental treatments, but we only broadcasted mobbing calls during the social and social + personal treatments to control for speaker presence. To control for human disturbance associated with presenting treatments, an experimenter approached the mount pole with a box and left without placing a mount during the social treatments. Thus, all estimated effects of different treatment categories above the observed effect during control treatments reflect responses to the biological components of the treatments. All treatments lasted 1 h to allow enough time for the chickadees to experience the treatment without habituating to it.

We used a stratified random design to assign treatments to feeders such that (i) a maximum of one treatment was conducted at a given feeder on any given experimental day and (ii) every experimental day consisted of each of the four treatments (social, personal, social + personal, control) being conducted across different feeders. A complete replicate required all four treatments to be carried out at each of the eight feeders. We began treatments at approximately 0930, 1100, 1230, and 1400 h, and we randomized the treatment order within an experimental day. We conducted treatments on every second day, with at least a 7‐day break between replicates to minimize any potential carryover and/or habituation effects. Thus, each replicate, wherein three 1 h predator presentations (one social, one personal, and one social + personal) and one 1 h control treatment occurred at any given feeder, occurred over 15 days (eight to complete the replicate and 7 days between subsequent replicates). We completed four replicates at each of the eight feeders over the course of the study.

To measure chickadee response to predation cues, we first filtered individual chickadees that were not detected at any feeder in the 1 h preceding the start of treatment, to increase the likelihood that birds included in analyses had experienced the treatment. We measured latency to resume feeding as the time it took an individual to return to a feeder following the start of a treatment. If a chickadee did not return to the feeder on that same day, we assigned them a maximum latency score as if they had returned at civil twilight. We calculated the post‐treatment feeding rate by multiplying the number of visits an individual made to a feeder during the first 20 min following treatment exposure by three, to reflect an hourly rate. We also measured the number of visits an individual made to a feeder in the hour preceding a treatment as a measure of their pre‐treatment (i.e., baseline) feeding rate.

### 2.3 Ethical Note

These experiments were carried out under permits from the Bird Banding Office in Canada for the catching and banding of chickadees (banding permit 10277 AK and 10277 AL), and from the University of Alberta Biosciences Animal Care and Use Committee (ACUC) for the use of animal subjects in experiments (permit AUP00002542 and AUP00002210). A Capture and Research permit from Alberta Fish and Wildlife (#56066, #56065, 19‐056) and a Canadian Wildlife Service Scientific permit from Environment Canada (#13‐ABSC004) were also obtained. We took several measures to minimize stress during capture and handling. We monitored mist nets continuously during capture attempts so that birds could be processed quickly, we did not attempt captures during inclement weather, we immediately released any birds that appeared unwell or stressed without processing them, birds were released after handling at the site of their initial capture, and all handling (banding, blood sampling, morphometric measurements) was completed in under 10 min. During initial capture, an experienced bird handler (Jan J. Wijmenga) collected a small (< 20 μL) blood sample from the brachial vein. We did not collect blood samples for individuals more than once since the samples were only needed for molecular sexing.

### 2.4 Statistical Analysis

We conducted all statistical analyses in the R statistical environment version 4.3.1 (R Core Team 2023) using the RStudio interface (Posit Team 2024). We constructed two multivariate models to explore the effect of daily ambient temperature on: (1) log‐transformed latency to resume feeding (by treatment) and (2) post‐treatment feeding rate (by treatment). Thus, each multivariate model had four response variables corresponding to each treatment (control, social, personal, and social + personal), each modeled with Gaussian error distributions using the “MCMCglm” package in R (Hadfield 2010). We included mean daily temperature and sex (−0.5 = M, 0.5 = F) as fixed effects as these were found to be important sources of variation in response to treatments in an earlier analysis (following Arteaga‐Torres et al. 2020). We mean‐centered and standardized treatment temperature at the level of the entire dataset prior to including it as a fixed effect in our analyses to facilitate comparison of effect between variables on different scales (Gelman 2008). Therefore, our model intercept estimates reflect estimated effects at the average treatment temperature across the study days (−11.21°C), and estimated effects of treatment temperature reflect the effect of 1 standard deviation (SD) change in temperature (8.13°C). A previous analysis of this dataset (Arteaga‐Torres et al. 2020) used the same scaling procedure, so we were able to verify the reproducibility of the earlier results. For our model of post‐treatment feeding rate, we additionally centered (mean = 55.02 min) and scaled (SD = 84.48 min) latency to resume feeding and included it as a covariate, as latency was previously shown to affect subsequent visit rates (see Arteaga‐Torres et al. 2020). We included individual ID as a random effect to control for nonindependence of repeated measures on the same individuals.

Initially, we used the same random effects structure as in the earlier (univariate) analysis of the dataset (Arteaga‐Torres et al. 2020) which additionally included feeder ID and replicate number. However, in our multivariate analyses, this resulted in poor model convergence, likely due to the relatively small variance attributable to feeder ID and replicate (see table 1 in Arteaga‐Torres et al. 2020). To ensure that excluding feeder ID and replicate number did not unduly influence results, we re‐ran the same univariate models presented in Arteaga‐Torres et al. (2020) both with and without feeder ID and replicate number as random effects. Excluding feeder ID and replicate number yielded qualitatively similar results and had no effect on biological interpretations compared to the models that included these random effects (see Table S1). Thus, models presented in the main text do not include either feeder ID or replicate number as random effects.

For both multivariate models presented here, we used an uninformative prior and verified that model results were robust to changes in prior specification, which they were (alternative prior specifications tested are available in the archived code for this paper (Reid et al. 2025)). Both models were run for 106,000 iterations with a burn‐in of 6000 and thinning of 100, so that 1000 estimates were retained for constructing posterior distributions. Visual inspection of posterior plots confirmed that models had converged and estimates were not temporally auto‐correlated. Changes in repeatability can arise via changes in within‐ and/or among‐individual variation, and similarly, changes in within‐ and/or among‐individual variation can lead to increased, decreased, or no change in repeatability (Dochtermann and Royauté 2019). Therefore, for completeness, we report among‐individual and within‐individual variance estimates (following Royauté and Dochtermann 2021), as well as the adjusted repeatability (following Nakagawa and Schielzeth 2010) separately for each treatment. Finally, we obtained the means of the posterior distribution of the covariance between responses (both within‐ and among‐individual covariances) using the “posterior.cor” function in the MCMCglmm package in R (Hadfield 2010).

Estimates are presented as the mode of the posterior distribution with the 95% credible interval (CrI). We used the 95% CrI to evaluate the level of support for a given effect. We describe 95% CrIs that did not overlap zero as providing strong support for an effect and estimates that were centered on zero as providing strong support for lack of an effect or no support for an effect. For estimates not centered on zero but whose 95% CrI overlapped zero, we calculated the proportion of estimates (pr) that were above (for negative mean estimates) or below (for positive mean estimates) zero to aid in the interpretation of the strength of support. For reference, an overlap of 0.20 corresponds to four times greater support (i.e., 0.80/0.20) for the interpretation of an effect in the reported direction compared to the interpretation of an effect in the opposing direction (Marsman and Wagenmakers 2017).

## 3 Results

### 3.1 Fixed Effects

Analyses of latency to resume feeding and post‐treatment feeding rate of chickadees in response to different cues of predation risk are presented elsewhere (Arteaga‐Torres et al. 2020). Our multivariate modeling approach—which treated responses to each treatment as separate traits and allowed us to address novel questions regarding covariance in response to different treatments (see below) – reproduced the key findings reported previously in Arteaga‐Torres et al. (2020) (Table 1). Specifically, the control treatment resulted in the lowest latency to resume feeding, the social treatment resulted in intermediate latencies, and the personal and social + personal treatments resulted in the longest latencies to resume feeding. Temperature had a positive effect on latency to resume feeding during the control, social, and social + personal treatments, and no effect during the personal treatment, and there was no effect of sex on latency to resume feeding (Table 1). The social treatment resulted in the lowest post‐treatment feeding rate, whereas feeding rates following control, personal, and social + personal treatments were similar (Table 2). Temperature had a negative effect on post‐treatment feeding rate during the social treatment and had no effect during all other treatments, and females had lower post‐treatment feeding rates following all treatments than males (Table 2).

**TABLE 1:** MCMCglmm model results for log latency to resume feeding (sec).

	Control	Social	Personal	Social + Personal
Fixed effects	β (95% CrI)	β (95% CrI)	β (95% CrI)	β (95% CrI)
Intercept^a^	6.63 (6.39, 6.82)	6.89 (6.64, 7.05)	7.79 (7.56, 7.96)	7.69 (7.49, 7.96)
Temperature^b^	0.20 (0.06, 0.33)	0.12 (−0.01, 0.36) pr = 0.04	0.07 (−0.1, 0.18) pr = 0.31	0.19 (0.02, 0.32)
Sex^c^	−0.24 (−0.64, 0.26) pr = 0.19	0.07 (−0.24, 0.58) pr = 0.22	−0.01 (−0.54, 0.31) pr = 0.33	0.32 (−0.31, 0.69) pr = 0.16
Random effects	*σ* (95% CrI)	*σ* (95% CrI)	*σ* (95% CrI)	*σ* (95% CrI)
ID	0.43 (0.18, 0.86)	0.11 (0.00, 0.42)	0.25 (0.12, 0.58)	0.77 (0.40, 1.24)
Residual	1.47 (1.14, 1.80)	2.28 (1.83, 2.70)	1.42 (1.18, 1.74)	1.30 (1.08, 1.67)
Repeatability^d^	*r* (95% CrI)	*r* (95% CrI)	*r* (95% CrI)	*r* (95% CrI)
ID‐Repeatability	0.25 (0.09, 0.39)	0.06 (0.00, 0.16)	0.18 (0.07, 0.30)	0.39 (0.23, 0.50)

*Note:* Latencies following each treatment were fitted as separate response variables and modeled with Gaussian errors as a function of temperature and sex. Proportion overlap (pr) values are reported for estimates with CrIs that overlap zero.

^a^ Intercept estimate is for an observation at the mean temperature (−11.21°C) in the dataset, controlling for sex differences.

^b^ Temperature was centered and scaled prior to analyses so that estimates reflect the effect of a 1 SD change in temperature (8.13°C) on the response variables.

^c^ Sex was coded as males = −0.5, females = 0.5 so that the estimated sex effect is the difference between males and females; but the intercept is estimated for the “averag” sex (i.e., 0).

^d^ Adjusted repeatability calculated following Nakagawa and Schielzeth (2010).

**TABLE 2:** MCMCglmm model results for post‐treatment feeding rate (visits/h).

	Control	Social	Personal	Social + Personal
Fixed effects	β (95% CrI)	β (95% CrI)	β (95% CrI)	β (95% CrI)
Intercept^a^	19.02 (15.94, 20.82)	16.73 (14.99, 18.32)	17.54 (16.06, 19.37)	18.20 (16.56, 19.57)
Temperature^b^	0.66 (−0.99, 1.54) pr = 0.35	−1.64 (−2.59, −0.47)	−0.84 (−2.26, 0.15) pr = 0.05	−0.45 (−1.44, 1.04) pr = 0.34
Sex^c^	−3.81 (−7.36, −0.76)	−4.08 (−6.47, −0.25)	−2.26 (−6.33, 0.83) pr = 0.08	−2.97 (−5.97, −0.03)
Latency^b^	−0.04 (−4.09, 4.32) pr = 0.49	2.68 (0.81, 3.86)	3.21 (1.12, 5.72)	5.86 (2.80, 7.72)
Random effects	*σ* (95% CrI)	*σ* (95% CrI)	*σ* (95% CrI)	*σ* (95% CrI)
ID	20.96 (7.09, 39.53)	18.83 (7.80, 41.02)	21.58 (5.58, 37.10)	9.53 (1.79, 25.71)
Residual	87.67 (74.66, 111.64)	69.47 (57.05, 85.79)	96.39 (79.85, 118.42)	92.71 (76.77, 114.53)
Repeatability^d^	*r* (95% CrI)	*r* (95% CrI)	*r* (95% CrI)	*r* (95% CrI)
ID‐Repeatability	0.18 (0.08, 0.32)	0.25 (0.10, 0.40)	0.17 (0.06, 0.30)	0.10 (0.01, 0.22)

*Note:* Feeding rates following each treatment were fitted as separate response variables and modeled with Gaussian errors as a function of temperature, sex, and latency to resume feeding. Proportion overlap (pr) values are reported for estimates with CrIs that overlap zero.

^a^ Intercept estimate is for an observation at the mean temperature (−11.21°C) and latency to resume feeding (3300.92 s/55.02 min) in the dataset, controlling for sex differences.

^b^ Temperature and latency to resume feeding were both centered and scaled prior to analyses so that estimates reflect the effect of a 1 SD change in temperature (8.13°C) or latency (5074.67 s/84.48 min) on the response variables.

^c^ Sex was coded as males = −0.5, females = 0.5 so that the estimated sex effect is the difference between males and females, but the intercept is estimated for the “averag” sex (i.e., 0).

^d^ Adjusted repeatability calculated following Nakagawa and Schielzeth (2010).

### 3.2 Variance Components and Repeatability

**FIGURE 1:**
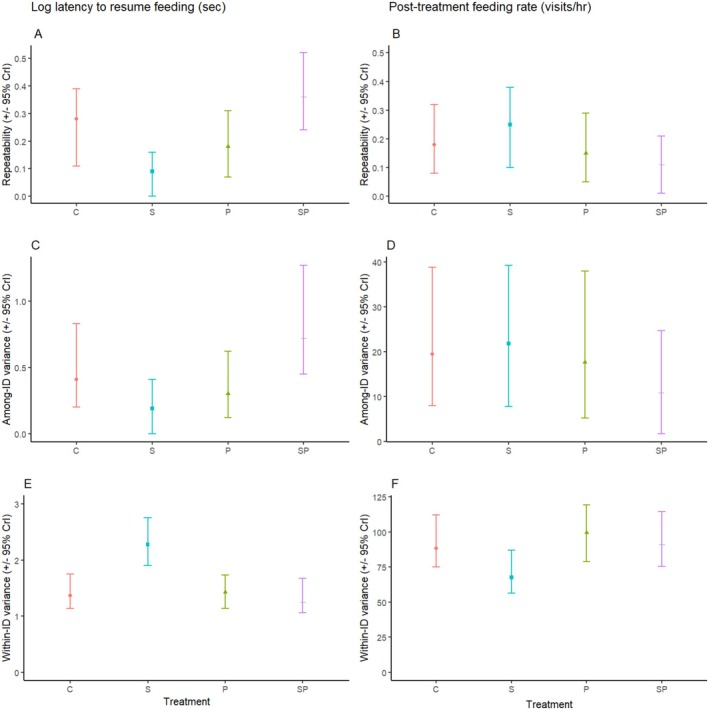
Adjusted repeatability (panels A, B), among‐individual variance (panels C, D), and within‐individual variance (panels E, F) in log latency to resume feeding (panels A, C, E) and post‐treatment feeding rates (panels B, D, F) for each treatment. Abbreviations have been used to identify treatments (i.e., C = control, *P* = personal information, S = social information, SP = social + personal information). Points represent posterior modes; error bars represent 95% credible intervals.

We observed treatment‐related differences in repeatability for both the log latency to resume feeding and post‐treatment feeding rate, resulting from a combination of treatment‐related differences in both among‐ and within‐individual variance (Tables 1 and 2, Figure 1). The repeatability of latency to resume feeding differed across treatments (see Table 3 for all pairwise comparisons). Repeatability was lowest following the social treatment, intermediate following both personal and control treatments, and highest following the social + personal treatment. These patterns mirrored those seen in among‐individual variance (Figure 1). Within‐individual variance in latency to resume feeding was similar for all treatment categories except the social treatment, which was significantly higher than all other treatments (Table 3).

**TABLE 3:** Comparisons of among‐individual, within‐individual, and repeatability estimates for latencies to resume feeding values (presented in Table 1), and post‐treatment feeding rate (presented in Table 2) for each pairwise comparison of treatments.

	Log latency to resume feeding (s)		Post‐treatment feeding rate (feeder visits/h)	
	β ± 95% CrI	pr	β ± 95% CrI	pr
Among‐individual variance				
C‐S	0.25 (−0.06, 0.68)	0.05	−0.44 (−24.74, 19.44)	0.44
C‐P	0.07 (−0.24, 0.53)	0.22	2.12 (−22.87, 21.68)	0.46
C‐SP	−0.24 (−0.85, 0.20)	0.09	7.65 (−9.59, 29.32)	0.15
S‐P	−0.25 (−0.46, 0.18)	0.16	−3.92 (−20.39, 24.73)	0.58
S‐SP	−0.56 (−1.09, −0.17)	0.01	6.64 (−10.22, 29.99)	0.11
P‐SP	−0.36 (−0.89, −0.04)	0.01	4.33 (−8.26, 27.38)	0.14
Within‐individual variance				
C‐S	−0.73 (−1.31, −0.35)	0.00	20.66 (−3.16, 46.24)	0.04
C‐P	−0.02 (−0.47, 0.41)	0.53	−0.79 (−36.13, 16.92)	0.31
C‐SP	0.29 (−0.34, 0.54)	0.30	−5.91 (−27.06, 26.83)	0.44
S‐P	0.90 (0.33, 1.38)	0.00	−25.12 (−54.09, −4.18)	0.02
S‐SP	1.04 (0.40, 1.42)	0.00	−23.99 (−46.26, 1.46)	0.03
P‐SP	0.19 (−0.27, 0.50)	0.33	2.99 (−19.97, 32.50)	0.35
Repeatability				
C‐S	0.19 (0.02, 0.33)	0.02	−0.02 (−0.26, 0.11)	0.28
C‐P	0.04 (−0.13, 0.22)	0.25	0.08 (−0.14, 0.18)	0.43
C‐SP	−0.15 (−0.33, 0.05)	0.10	0.08 (−0.11, 0.21)	0.15
S‐P	−0.10 (−0.25, 0.02)	0.06	0.10 (−0.10, 0.26)	0.23
S‐SP	−0.29 (−0.45, −0.14)	0.00	0.13 (−0.03, 0.31)	0.06
P‐SP	−0.18 (−0.35, −0.03)	0.01	0.03 (−0.07, 0.22)	0.17

*Note:* Abbreviations have been used to identify treatments (i.e., C = control, *P* = personal information, S = social information, SP = social + personal information).

Treatment‐related differences in the repeatability of post‐treatment feeding rates were less pronounced, with only the social + personal treatment having a lower repeatability than all other treatments (Table 3). Again, the patterns in repeatability largely mirrored patterns in among‐individual variance (Figure 1). As with latency to resume feeding, within‐individual variance for post‐treatment feeding rate differed only for the social treatment; however, in this case, it was lower compared to all other treatments (Table 3).

### 3.3 Covariance

**FIGURE 2:**
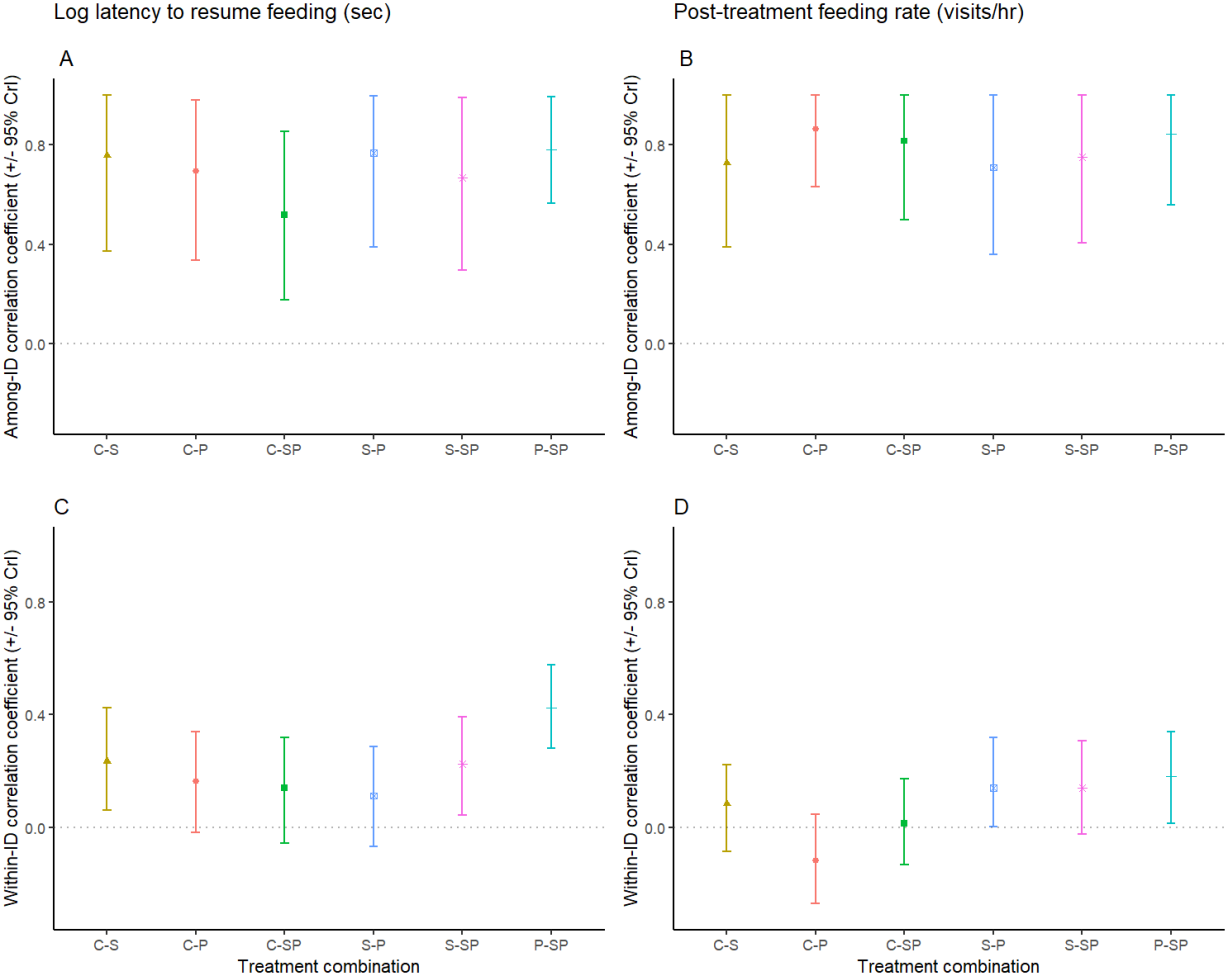
Among‐individual (panels A, B) and within‐individual (panels C, D) covariances between log latency to resume feeding (panels A, C) and post‐treatment feeding rates (panels B, D) for each pairwise combination of treatments. Abbreviations have been used to identify treatments (i.e., C = control, *P* = personal information, S = social information, SP = social+personal information). The dotted horizontal line at zero denotes no correlation. Correlations were obtained from the multivariate models using the posterior.cor function. Estimates and 95% CrI are also presented in Tables S2 and S3.

Finally, we were interested in covariation in responses across treatments. At both the within‐ and among‐individual levels, we observed positive correlations between all pairwise treatment combinations for latency to resume feeding (Figure 2, Table S2). Correlations were stronger at the among‐individual level (range: *r* = 0.53–0.78) compared to the within‐individual level (range: *r* = 0.11–0.43). This is not surprising since these correlations would reflect common factors shaping responses within replicates, and replicates explained little variance in either of our response variables (see Table S1). Additionally, there was no evidence that among‐individual correlations were higher between treatments with shared information sources (Figure 2).

We also observed strong positive correlations between all pairwise treatment combinations for post‐treatment feeding rates at the among‐individual level (range: *r* = 0.70–0.84), but not at the within‐individual level (range: *r* = −0.12–0.18) (Figure 2, Table S3). Again, there was no evidence that among‐individual correlations were higher between treatments with shared information sources (Figure 2).

## 4 Discussion

We re‐analysed data from a previous study on the effects of perceived predation risk on latency to resume feeding and post‐treatment feeding rate in a wild population of black‐capped chickadees (Arteaga‐Torres et al. 2020) to ask whether individuals differ in how they respond to personal versus social information about predation risk. Contrary to our prediction, we did not find evidence of correlations in response to particular cue modalities (social, personal, and social + personal). However, we observed that different treatments elicited different effects on among‐ and within‐individual response variance. Specifically, the social + personal information cue resulted in the highest among‐individual variance in latency to resume feeding, whereas the social information cue resulted in the lowest among‐individual variance and highest within‐individual variance in latency to resume feeding. Cue modality had less of an influence on the post‐treatment feeding rate variance components. Below, we discuss the implications of our results in terms of understanding the causes and consequences of individual variation in response to predation risk cues.

We first evaluated support for repeatable among‐individual variation in response to social versus personal cues about predation risk. Several earlier studies have shown that individuals differ repeatably in how they value social versus personal information in contexts such as foraging and nest building (Rieucau and Giraldeau 2011; Whittaker et al. 2023). Given that among‐individual differences in vulnerability to predation are well documented (reviewed in Mesa et al. 1994; Pettorelli et al. 2011), we expected that individuals should also differ in the costs and benefits of acquiring and responding to personal versus social information about that risk. Contrary to our prediction, we found no evidence that individuals show repeatable variation in their relative response to social (mobbing calls) versus personal (predator mount) information about predation risk. Among‐individual correlations between latency to resume feeding in response to different treatments were largely positive and broadly overlapped, indicating that individuals that responded strongly to one treatment also responded strongly to other treatments, irrespective of the information type. This finding is consistent with state‐dependent responses to predation risk and suggests that individuals differ consistently in the costs and/or benefits of delaying feeding in response to perceived predation threats. For example, within populations, individuals often exhibit marked and repeatable differences in energy requirements (Burton et al. 2011; Nespolo and Franco 2007), which may influence the extent to which they can delay foraging. In this case, chickadees with higher energy requirements may be expected to return to foraging sooner, regardless of perceived predation risk or information source.

To test whether energy requirements influenced antipredator response in our study, we evaluated, post hoc, whether foraging rates prior to the experiment (a proxy for baseline energy requirements) were negatively related to latency to resume feeding (see Table S4). We indeed found that chickadees with higher baseline feeding rates had significantly shorter latencies to resume feeding (Table S4). However, even after controlling for variation in baseline feeding, we still observed significant positive among‐individual correlations between responses to all treatment types (see Table S5), indicating that individual differences in energetic requirements cannot fully account for the correlated responses at the among‐individual level. This is consistent with the results of an earlier meta‐analysis showing that resting metabolic rate (a proxy for individual energetic requirements) explains part, but not all, of the repeatable individual variation in risk‐taking and antipredator behavior (reviewed in Mathot et al. 2019).

We also found that post‐treatment feeding rates did not differ as a function of treatment (Table 2) and were positively correlated at the among‐individual level (Table 2). This suggests that post‐treatment feeding rate is not a response to the type of predator cue presented but instead may reflect individual energy requirements. Consistent with this, our post hoc analyses, which included pre‐treatment feeding rate as a fixed effect, showed that chickadees with higher baseline feeding rates also had higher post‐treatment feeding rates (Table S6). However, similar to the latency to resume feeding response (discussed above), even after controlling for variation in baseline feeding rates, we still observed significant positive among‐individual correlations between post‐treatment foraging rates (see Table S7), indicating that individual differences in energetic requirements cannot fully account for the correlated responses in post‐treatment feeding rates at the among‐individual level.

Our multivariate analyses reproduced previously reported results for mean responses (log latency to resume feeding and post‐treatment feeding rate) across treatments (see Arteaga‐Torres et al. (2020) and Tables 1 and 2). However, modeling responses to each treatment as separate response variables allowed us to estimate treatment‐specific variance components, which generated two novel insights. First, we found that within‐individual variance in latency to resume feeding was highest following the social treatment on its own compared to the other treatments (Table 1, Figure 1). This result is consistent with the notion that social cues may be less reliable sources of information than personal cues, such as observing a predator. Specifically, there is evidence that mobbing calls can be given as false alarms (Møller 1988; Munn 1986), which could reduce an individual's likelihood of responding if, by responding, the individual forgoes foraging opportunities.

Second, we found that the social + personal treatment resulted in the highest among‐individual variance in latency to resume feeding relative to the other three treatment categories (Table 1, Figure 1). This suggests that chickadees may vary in how they integrate personal and social information when they are received simultaneously. Different rules for the integration of social and personal information have been found across taxa where species may integrate cues synergistically (i.e., combining cues elicits a larger response than either would alone), redundantly (the same response), or antagonistically (a smaller response) (reviewed in Mathot et al. 2024; Munoz and Blumstein 2012). Our finding that individuals varied most in their latency to resume feeding following the treatment with both social and personal information cues suggests that variation in cue integration may exist within our population. Our results suggest that some individuals experienced the social + personal treatment as a higher perceived predation risk than either cue alone, potentially because combined predator cues provide higher certainty of predation risk (Munoz and Blumstein 2012; Weissburg et al. 2014), whereas others perceived it as less threatening, potentially because mobbing calls can suggest that the threat is being “attended to” (Arteaga‐Torres et al. 2020). Although it is unclear why chickadees in our population might differ in how they integrate cues, variation in predation levels experienced by individuals in early life (e.g., Beaty et al. 2016; Brown et al. 2006; Feyten et al. 2021; Winandy et al. 2021; Wisenden et al. 2024), as well as predation levels experienced by mothers (Beaty et al. 2016; Winandy et al. 2021) have been shown to influence among‐individual differences in antipredatory response. Furthermore, younger individuals may be more likely to respond to alarm calls because they have not learned the optimal degree of response yet, or because they are intrinsically more vulnerable to predation (Hollen and Radford 2009). Thus, we suggest that among‐individual differences in cue integration rules may result from differences in prior experience. Additionally, while the social + personal treatment was associated with the highest among‐individual variation in latency to resume feeding, it was also associated with the lowest among‐individual variation in post‐treatment feeding rate (Table 2, Figure 1). This suggests that larger differences in foraging delays resulted in smaller differences in energetic state once foraging did resume, and hints that variation in energetic state might also have shaped the information integration strategy adopted by chickadees in this study. To our knowledge, this is the first study that has found among‐individual differences in cue integration, and further studies are needed to directly assess the factors that might influence among‐individual differences in cue integration within animal populations.

### 4.1 Conclusion

We found that while, at the population level, chickadees exhibited differences in mean response to different sources of information about predation risk, at the among‐individual level, responses were strongly positively correlated across all treatment combinations. This suggests that state‐dependent costs and/or benefits of predation risk are a major driver of among‐individual differences in response rather than differences in preference for personal and social information. However, we observed differences in among‐individual variance across treatments, suggesting that individual chickadees do differ in how they integrate personal and social information about predation risk. Specifically, the social + personal information cue resulted in the highest among‐individual variance in latency to resume feeding, which may reflect individual differences in information integration rules (e.g., synergistic, redundant, antagonistic). We suggest that among‐individual differences in energetic state and/or prior experience with predation risk may shape information integration rules in chickadees; however, future work is needed to test these ideas.

## Author Contributions

**Emma L. C. Reid:** conceptualization (equal), formal analysis (equal), visualization (equal), writing – original draft (lead), writing – review and editing (equal). **Megan LaRocque:** conceptualization (equal), formal analysis (equal), supervision (equal), visualization (equal), writing – original draft (supporting), writing – review and editing (equal). **Josue David Arteaga‐Torres:** investigation (lead), writing – review and editing (equal). **Kimberley J. Mathot:** conceptualization (equal), formal analysis (supporting), funding acquisition (lead), supervision (equal), writing – original draft (supporting), writing – review and editing (equal).

## Conflicts of Interest

The authors declare no conflicts of interest.

## Supporting information


**Data S1:** ece372016‐sup‐0001‐Supinfo.docx.

## Data Availability

All data and code required to reproduce results and figures presented in the manuscript are available here: https://osf.io/bhk23 (Reid et al. 2025).
