# Supplementary material for: Individual Antipredator Responses Are Positively Correlated Across Cue Types in Free‐Living Black‐Capped Chickadees ( Poecile atricapillus )
Source: Ecol Evol. 2025 Aug 17;15(8):e72016. doi: 10.1002/ece3.72016 (PMC12358306; doi:10.1002/ece3.72016)
Supplement: Supplementary file 1 — Data S1: ece372016‐sup‐0001‐Supinfo.docx. [file ECE3-15-e72016-s001.docx]

**Electronic Supplementary Material for: Individual antipredator responses are positively correlated across cue types in free-living black-capped chickadees (*Poecile atricapillus*)**

Authors names redacted for review.

Contents

[Table S1. Comparison of LMM model results for latency to resume feeding and post-treatment feeding rate, using the original model from Arteaga-Torres et al. (2020) and a simplified model excluding feeder ID and replicate number as random effects. 3](#_Toc189736955)

[Table S2. Within- and among-individual covariance matrix for latency to resume feeding. Values are reported as correlation coefficients with 95% credible intervals. Values above the diagonal are among-individual correlations, and values below the diagonal are within-individual correlations. 4](#_Toc189736956)

[Table S3. Within- and among-individual covariance matrix for post-treatment feeding rate. Values are reported as correlation coefficients with 95% credible intervals. Values above the diagonal are among-individual correlations, and values below the diagonal are within-individual correlations. 5](#_Toc189736957)

[Table S4. MCMCglmm model results for latency to resume feeding, including baseline feeding rate as a fixed effect. Following each treatment, latencies were log-transformed, fitted as separate response variables, and modelled with gaussian errors as a function of baseline feeding rate, temperature, sex, and latency to resume feeding. 6](#_Toc189736958)

[Table S5. Within- and among-individual covariance matrix for estimates derived from model of latency to resume feeding presented in Table S4. Values are reported as correlation coefficients with 95% credible intervals. Values above the diagonal are among-individual correlations, and values below the diagonal are within-individual correlations. 7](#_Toc189736959)

[Table S6. MCMCglmm model results for post-treatment feeding rate, including baseline feeding rate as a fixed effect. Feeding rates following each treatment were fitted as separate response variables and modelled with gaussian errors as a function of baseline feeding rate, temperature, sex, and latency to resume feeding. 8](#_Toc189736960)

[Table S7. Within- and among-individual covariance matrix for estimates derived from model of post-treatment feeding rate presented in Table S6. Values are reported as correlation coefficients with 95% credible intervals. Values above the diagonal are among-individual correlations, and values below the diagonal are within-individual correlations. 9](#_Toc189736961)

[References 10](#_Toc189736962)

# Table S1. Comparison of LMM model results for latency to resume feeding and post-treatment feeding rate, using the original random effects structure from Arteaga-Torres et al. (2020), and a simplified model excluding feeder ID and replicate number as random effects.

|  | **Latency to Resume Feeding (sec)** | | **Feeding Rate (visits h^−1^)** | |
| --- | --- | --- | --- | --- |
|  | **Original** | **Simplified** | **Original** | **Simplified** |
| ***Fixed Effects*** | ***β (95% CrI)*** | ***β (95% CrI)*** | ***β (95% CrI)*** | ***β (95% CrI)*** |
| Sex | 0.01 (−0.27, 0.33) | 0.08 (-0.24, 0.43) | −3.38 (−5.82, −1.14) | -3.31 (-5.52, -1.14) |
| Treatment |  |  |  |  |
| Control | 6.49 (6.01, 6.84) | 6.46 (6.28, 6.69) | 19.61 (17.51, 22.16) | 19.58 (18.01, 20.99) |
| Acoustic | 6.64 (6.23, 7.04) | 6.71 (6.48, 6.90) | 16.39 (14.12, 18.77) | 16.08 (14.75, 17.77) |
| Visual | 7.55 (7.15, 7.96) | 7.68 (7.41, 7.85) | 17.11 (14.67, 19.31) | 17.12 (15.70, 18.68) |
| Acoustic + Visual | 7.43 (7.02, 7.85) | 7.50 (7.27, 7.70) | 17.11 (14.91, 19.70) | 17.34 (15.44, 18.50) |
| Temperature |  |  |  |  |
| by Control | 0.22 (0.06, 0.34) | 0.27 (0.09, 0.38) | 0.14 (−1.05, 1.28) | -0.18 (-1.36, 0.95) |
| by Acoustic | 0.23 (0.08, 0.36) | 0.26 (0.09, 0.38) | −1.49 (−2.85, −0.42) | -1.72 (-2.84, -0.35) |
| by Visual | 0.09 (−0.08, 0.21) | 0.03 (-0.11, 0.17) | −0.86 (−2.14, 0.28) | -1.14 (-2.05, 0.35) |
| by Acoustic + Visual | 0.18 (0.06, 0.37) | 0.20 (0.03, 0.34) | −0.08 (−1.46, 1.18) | -0.21 (-1.47, 0.99) |
| LRF | N/A | N/A | 1.96 (1.32, 2.63) | 1.66 (1.21, 2.51) |
| ***Random Effects*** | ***σ (95% CrI)*** | ***σ (95% CrI)*** | ***σ (95% CrI)*** | ***σ (95% CrI)*** |
| ID | 0.33 (0.19, 0.50) | 0.37 (0.25, 0.58) | 17.09 (9.36, 25.12) | 13.67 (9.28, 24.43) |
| Feeder | 0.12 (0.03, 0.57) | N/A | 0.09 (0.00, 0.24) | N/A |
| Replicate | 0.00 (0.00, 0.07) | N/A | 3.78 (0.00, 13.66) | N/A |
| Residual | 1.23 (1.14, 1.38) | 1.29 (1.17, 1.43) | 87.0 (78.14, 95.01) | 88.00 (79.92, 96.08) |
| ***Repeatability*** | ***r (95% CrI)*** | ***r (95% CrI)*** | ***r (95% CrI)*** | ***r (95% CrI)*** |
| ID-Repeatability | 0.21 (0.14, 0.30) | 0.24 (0.16, 0.31) | 0.16 (0.10, 0.24) | 0.15 (0.10, 0.22) |

# Table S2. Within- and among-individual covariance matrix for latency to resume feeding. Values are reported as correlation coefficients with 95% credible intervals. Values above the diagonal are among-individual correlations, and values below the diagonal are within-individual correlations.

|  | Control | Acoustic | Visual | Acoustic+visual |
| --- | --- | --- | --- | --- |
| Control | - | 0.75 (0.26, 0.99) | 0.70 (0.31, 0.98) | 0.53 (0.19, 0.84) |
| Acoustic | 0.24 (0.06, 0.41) | - | 0.75 (0.25, 0.98) | 0.67 (0.20, 0.96) |
| Visual | 0.16 (-0.02, 0.33)  pr = 0.05 | 0.11 (-0.07, 0.29)  pr = 0.13 | - | 0.78 (0.52, 0.97) |
| Acoustic+visual | 0.14 (-0.07, 0.31)  pr = 0.09 | 0.22 (0.03, 0.40) | 0.43 (0.26, 0.56) | - |

# Table S3. Within- and among-individual covariance matrix for post-treatment feeding rate. Values are reported as correlation coefficients with 95% credible intervals. Values above the diagonal are among-individual correlations, and values below the diagonal are within-individual correlations.

|  | Control | Acoustic | Visual | Acoustic+visual |
| --- | --- | --- | --- | --- |
| Control | - | 0.72 (0.29, 0.98) | 0.85 (0.53, 0.99) | 0.81 (0.35, 0.99) |
| Acoustic | 0.09 (-0.07, 0.25)  pr = 0.14 | - | 0.70 (0.25, 0.98) | 0.75 (0.29, 0.99) |
| Visual | -0.12 (-0.28, 0.05)  pr = 0.08 | 0.13 (-0.02, 0.28)  pr = 0.04 | - | 0.84 (0.44, 1.00) |
| Acoustic+visual | 0.02 (-0.13, 0.16)  pr = 0.41 | 0.13 (-0.04, 0.31)  pr = 0.06 | 0.18 (0.01, 0.34) | - |

# Table S4. MCMCglmm model results for latency to resume feeding, as a function of baseline feeding rate, temperature, and sex. Latencies following each treatment were log-transformed fitted as separate response variables, and modelled with Gaussian errors.

|  | **Control** | **Acoustic** | **Visual** | **Acoustic + Visual** |
| --- | --- | --- | --- | --- |
| ***Fixed Effects*** | ***β (95% CrI)*** | ***β (95% CrI)*** | ***β (95% CrI)*** | ***β (95% CrI)*** |
| Intercept^1^ | 6.72 (6.49, 6.92) | 6.90 (6.73, 7.16) | 7.86 (7.62, 8.06) | 7.69 (7.51, 8.05) |
| Temperature^2^ | 0.28 (0.03, 0.61) | 0.18 (-0.16, 0.60) | -0.05 (-0.31, 0.25) | 0.28 (0.00, 0.58) |
| Baseline Feeding Rate^2^ | -0.65 (-0.98, -0.26) | -0.59 (-1.04, -0.18) | -0.60 (-0.86, -0.21) | -0.40 (-0.71, -0.01) |
| Sex^3^ | -0.16 (-0.61, 0.21) | -0.08 (-0.38, 0.47) | -0.10 (-0.54, 0.22) | 0.27 (-0.25, 0.76) |
| ***Random Effects*** | ***σ (95% CrI)*** | ***σ (95% CrI)*** | ***σ (95% CrI)*** | ***σ (95% CrI)*** |
| ID | 0.33 (0.11, 0.68) | 0.12 (0.00, 0.37) | 0.23 (0.07, 0.50) | 0.76 (0.40, 1.20) |
| Residual | 1.40 (1.12, 1.73) | 2.21 (1.79, 2.63) | 1.27 (1.10, 1.65) | 1.34 (1.00, 1.61) |
| ***Repeatability^4^*** | ***r (95% CrI)*** | ***r (95% CrI)*** | ***r (95% CrI)*** | ***r (95% CrI)*** |
| ID-Repeatability | 0.17 (0.07, 0.35) | 0.06 (0.00, 0.15) | 0.14 (0.04, 0.27) | 0.36 (0.23, 0.51) |

1. The intercept estimate is for an observation at the mean temperature (-11.21°C) and mean baseline feeding rate (8.16 visits/hr) in the dataset, controlling for sex differences.

2. Temperature and baseline feeding rate were centered and scaled prior to analyses so that estimates reflect the effect of 1 s.d. change in temperature or baseline feeding rate on the response variables.

3. Sex was coded as males = -0.5, females = 0.5 so that the estimated sex effect is the difference between males and females, but the intercept is estimated for the ‘average’ sex (i.e., 0).

4. Adjusted repeatability calculated following Nakagawa and Schielzeth (2010).

# Table S5. Within- and among-individual covariance matrix for estimates derived from model of latency to resume feeding presented in Table S4. Values are reported as correlation coefficients with 95% credible intervals. Values above the diagonal are among-individual correlations, and values below the diagonal are within-individual correlations.

|  | Control | Acoustic | Visual | Acoustic + Visual |
| --- | --- | --- | --- | --- |
| Control | - | 0.74 (0.24, 0.98) | 0.69 (0.22, 0.98) | 0.49 (0.09, 0.84) |
| Acoustic | 0.22 (0.04, 0.38) | - | 0.76 (0.24, 0.99) | 0.66 (0.19, 0.96) |
| Visual | 0.14 (-0.05, 0.31) | 0.12 (-0.05, 0.28) | - | 0.75 (0.42, 0.96) |
| Acoustic + Visual | 0.09 (-0.11, 0.29) | 0.17 (0.00, 0.35) | 0.39 (0.23, 0.55) | - |

# Table S6. MCMCglmm model results for post-treatment feeding rate, as a function of baseline feeding rate, temperature, and sex, . Feeding rates following each treatment were fitted as separate response variables and modelled with Gaussian errors.

|  | **Control** | **Acoustic** | **Visual** | **Acoustic + Visual** |
| --- | --- | --- | --- | --- |
| ***Fixed Effects*** | ***β (95% CrI)*** | ***β (95% CrI)*** | ***β (95% CrI)*** | ***β (95% CrI)*** |
| Intercept^1^ | 18.11 (16.41, 19.63) | 15.62 (14.16, 17.41) | 16.83 (15.01, 18.67) | 17.44 (15.98, 19.19) |
| Temperature^2^ | 0.97 (-1.93, 3.17) | -2.46 (-4.38, 0.11) | -1.44 (-4.39, 0.65) | -0.01 (-2.60, 2.50) |
| Baseline Feeding Rate^2^ | 4.27 (0.74, 6.66) | 2.19 (0.09, 5.10) | 3.66 (1.29, 7.04) | 0.97 (-2.47, 3.92) |
| Sex^3^ | -3.56 (-6.71, -0.82) | -2.87 (-5.96, 0.22) | -2.36 (-4.96, 1.60) | -2.57 (-5.44, 0.56) |
| ***Random Effects*** | ***σ (95% CrI)*** | ***σ (95% CrI)*** | ***σ (95% CrI)*** | ***σ (95% CrI)*** |
| ID | 10.49 (1.87, 30.70) | 20.37 (5.87, 38.67) | 22.24 (4.54, 37.47) | 2.87 (0.00, 20.36) |
| Residual | 93.98 (76.68, 112.45) | 74.21 (59.28, 90.04) | 94.82 (78.78, 117.26) | 95.92 (80.29, 122.71) |
| ***Repeatability^4^*** | ***r (95% CrI)*** | ***r (95% CrI)*** | ***r (95% CrI)*** | ***r (95% CrI)*** |
| ID-Repeatability | 0.11 (0.02, 0.26) | 0.23 (0.08, 0.37) | 0.17 (0.05, 0.30) | 0.05 (0.00, 0.18) |

1. The intercept estimate is for an observation at the mean temperature (-11.21°C) and mean baseline feeding rate (8.16 visits/hr) in the dataset, controlling for sex differences.

2. Temperature and baseline feeding rate were centered and scaled (by dividing by 2 s.d.) prior to analyses so that estimates reflect the effect of 1 s.d. change in temperature or baseline feeding rate on the response variables.

3. Sex was coded as males = -0.5, females = 0.5 so that the estimated sex effect is the difference between males and females, but the intercept is estimated for the ‘average’ sex (i.e., 0).

4. Adjusted repeatability calculated following Nakagawa and Schielzeth (2010).

# Table S7. Within- and among-individual covariance matrix for estimates derived from model of post-treatment feeding rate presented in Table S6. Values are reported as correlation coefficients with 95% credible intervals. Values above the diagonal are among-individual correlations, and values below the diagonal are within-individual correlations.

|  | Control | Acoustic | Visual | Acoustic + Visual |
| --- | --- | --- | --- | --- |
| Control | - | 0.63 (0.10, 0.98) | 0.80 (0.37, 0.99) | 0.69 (-0.18, 0.99) |
| Acoustic | 0.07 (-0.09, 0.22) | - | 0.64 (0.18, 0.98) | 0.66 (-0.02, 0.98) |
| Visual | -0.09 (-0.25, 0.06) | 0.15 (-0.02, 0.31) | - | 0.75 (0.07, 0.99) |
| Acoustic + Visual | 0.02 (-0.12, 0.18) | 0.14 (-0.04, 0.32) | 0.17 (-0.01, 0.34) | - |

# References

Arteaga-Torres, J. D., Wijmenga, J. J., & Mathot, K. J. (2020). Visual cues of predation risk outweigh acoustic cues: a field experiment in black-capped chickadees. *Proceedings of the Royal Society B-Biological Sciences, 287*(1936). doi: 10.1098/rspb.2020.2002

Nakagawa, S., & Schielzeth, H. (2010). Repeatability for Gaussian and non-Gaussian data: a practical guide for biologists. *Biological Reviews, 85*(4), 935-956. doi: 10.1111/j.1469-185X.2010.00141.x
